# MR imaging-based risk stratification scoring system to predict clinical outcomes in carotid body tumors

**DOI:** 10.3389/fonc.2023.1200598

**Published:** 2024-01-22

**Authors:** Abhishek Mahajan, Atif Shaikh, Shreya Shukla, Richa Vaish, Ujjwal Agarwal, Vasundhara Smriti, Shivam Rastogi, Shonal Deokar, Shubham Suryavanshi, Pankaj Chaturvedi, Sarbani Ghosh Laskar, Kumar Prabhash, Vijay Patil, Vanita Noronha, Nandini Menon, Prathamesh Pai, Gouri Pantvaidya, Swapnil Ulhas Rane, Munita Bal, Neha Mittal, Asawari Patil, Anil Keith Dcruz

**Affiliations:** ^1^ Department of Imaging, The Clatterbridge Cancer Centre NHS Foundation Trust, Liverpool, United Kingdom; ^2^ Faculty of Health and Life Sciences, University of Liverpool, Liverpool, United Kingdom; ^3^ Department of Radiology, Tata Memorial Hospital, Mumbai, India; ^4^ Department of Head and Neck Surgical Oncology, Tata Memorial Hospital, Mumbai, India; ^5^ Department of Radiation Oncology, Tata Memorial Hospital, Mumbai, India; ^6^ Department of Medical Oncology, Tata Memorial Hospital, Mumbai, India; ^7^ Department of Pathology, Tata Memorial Hospital, Mumbai, India

**Keywords:** carotid body tumor, paraganglioma, magnetic resonance imaging, Shamblin classification, angle of contact, distance from skull base, scoring

## Abstract

**Objectives:**

This study aims to evaluate the role of pretherapy MRI in predicting outcomes in carotid body tumors and propose a grading system for high- and low-risk characteristics.

**Materials and methods:**

A retrospective observational study of 44 patients with 51 lesions was carried out from year 2005 to 2020. MR images were reviewed for characteristics of carotid body tumor, and a score was given that was correlated with intra- and postoperative findings. The various other classifications and our proposed Mahajan classification were compared with Shamblin’s classification. The area under the curve and ROC curves were used to present the accuracy of different predictive models.

**Results:**

Our scoring system allotted a score of 0 to 15 on the basis of MRI characteristics, with scores calculated for patients in our study ranging from 0 to 13. Lesions with scores of 0–6 were considered low risk (45%), and scores of 7–15 were regarded as high risk for surgery (55%). The Mahajan classification stages tumors into four grades: I (10%), II (20%), IIIa (8%), and IIIb (62%). The frequency of vascular injury was 50% in category I and 64% in category IIIb. The frequency of cranial nerve injury was 50%, 66%, and 27% in categories I, II, and IIIb.

**Conclusion:**

The Mahajan classification of CBTs evaluates high-risk factors like the distance of the tumor from the skull base and the angle of contact with ICA, which form the major predictors of neurovascular damage and morbidity associated with its surgery. Though the Shamblin classification of CBT is the most widely accepted classification, our proposed Mahajan classification system provides an imaging-based alternative to prognosticate surgical candidates preoperatively.

## Highlights

- Carotid body tumors are common head and neck paragangliomas requiring surgical resection in symptomatic patients.
- Size, volume, distance from the skull base, and angle of contact with adjacent carotid vessels form the major predictors of neurovascular damage and morbidity associated with carotid body surgery.
- MRI can adequately assess tumor characteristics preoperatively to predict surgical outcomes and perioperative complications.
- MRI can be used as an effective prognostication tool with the use of the Mahajan classification and risk scoring system.

## Introduction

Paragangliomas are tumors that develop from paraganglion cells (1). They appear in the carotid space, jugular foramen, middle ear, and along the course of the vagus nerve, among other places (2). The most prevalent head and neck paragangliomas are carotid body tumors (3). Carotid body tumors (CBT), also known as chemodectomas, grow from the chemoreceptors found at the carotid bifurcation. Clinically, they present as painless, slow-growing lumps (4). Paraganglioma appears as a well-defined, hypoechoic mass at the carotid bifurcation on ultrasonography, with substantial vascularity on color Doppler (5). Internal flow voids can be seen on magnetic resonance imaging (MRI) in a T1 hypointense and T2 isointense to hyperintense mass (6). A computed tomography (CT) scan is used as the modality of choice for assessing bone involvement (7). Positron emission tomography (PET) imaging is useful for monitoring patients with malignant paragangliomas (8). The diagnosis, preoperative work-up, and surgical planning of these tumors are frequently done using imaging techniques (9). Carotid paragangliomas can be treated with surgery, radiation, or stereotactic radiosurgery (10). Because of the tumor’s high vascularity, proximity to the carotid vessels, local involvement of cranial nerves, and risk of stroke, surgery can be difficult (11). Large, unresectable tumors and multicentric illnesses benefit from radiotherapy (12). Surveillance with serial imaging to assess the growth of the tumor is recommended (13). For the satisfactory surgical outcome of these lesions, meticulous preoperative planning and cautious patient selection are required. Classification by Shamblin et al. for assessment of the resectability of these tumors is popularly used as a predictor of vascular morbidity. Shamblin classification divides them into three grades depending upon the extent of involvement of carotid vessels (14). Its modification by Luna-Ortiz et al. divides the third group into two further groups (15, 16).

The three classes under the Shamblin classification (14) include:

Class I: tumor localized with minimal vascular attachment, easily resectable;
Class II: tumor adherent or partially surrounding carotids; and
Class III: tumor intimately surrounding or encasing carotids.

The modified Shamblin classification by Luna-Ortiz et al. (15) further categorizes class III into class IIIa—tumor intimately surrounding or encasing carotids—and class IIIb—tumor partially or completely infiltrating vessel wall. While the Shamblin classification describes carotid vessel involvement, it cannot predict the occurrence of other complications (17). Our modification proposes to calculate the angle of contact using non-invasive cross- sectional imaging and additionally the distance of the tumor from the skull base. The study also evaluates the role of pretherapy MRI in predicting outcomes in CBTs by proposing a scoring system to stratify patients into high- and low-risk categories for surgery. Since the proposal of this study, newer classifications have also been published in the literature, which included variables included in our study and verified our thought process. These include the Peking Union Medical College Hospital (PUMCH) classification (types I to V) by Gu et al., who have classified them on the basis of carotid arterial encasement and vertical extension of the tumors on preoperative imaging (18). Another study by Jasper et al. proposed a scoring system based on parameters like tumor volume, the angle of contact, the presence of a peritumoral tuft of veins, and the loss of tumor adventitia interface on CT (19). Literature shows various classifications for CBTs, but very few studies have reported the role of MRI in predicting immediate and long-term outcomes. The purpose of our retrospective, single-center study was to redefine the objective criteria for MRI and predict surgical outcomes in these tumors.

## Materials and methods

**Figure 1:**
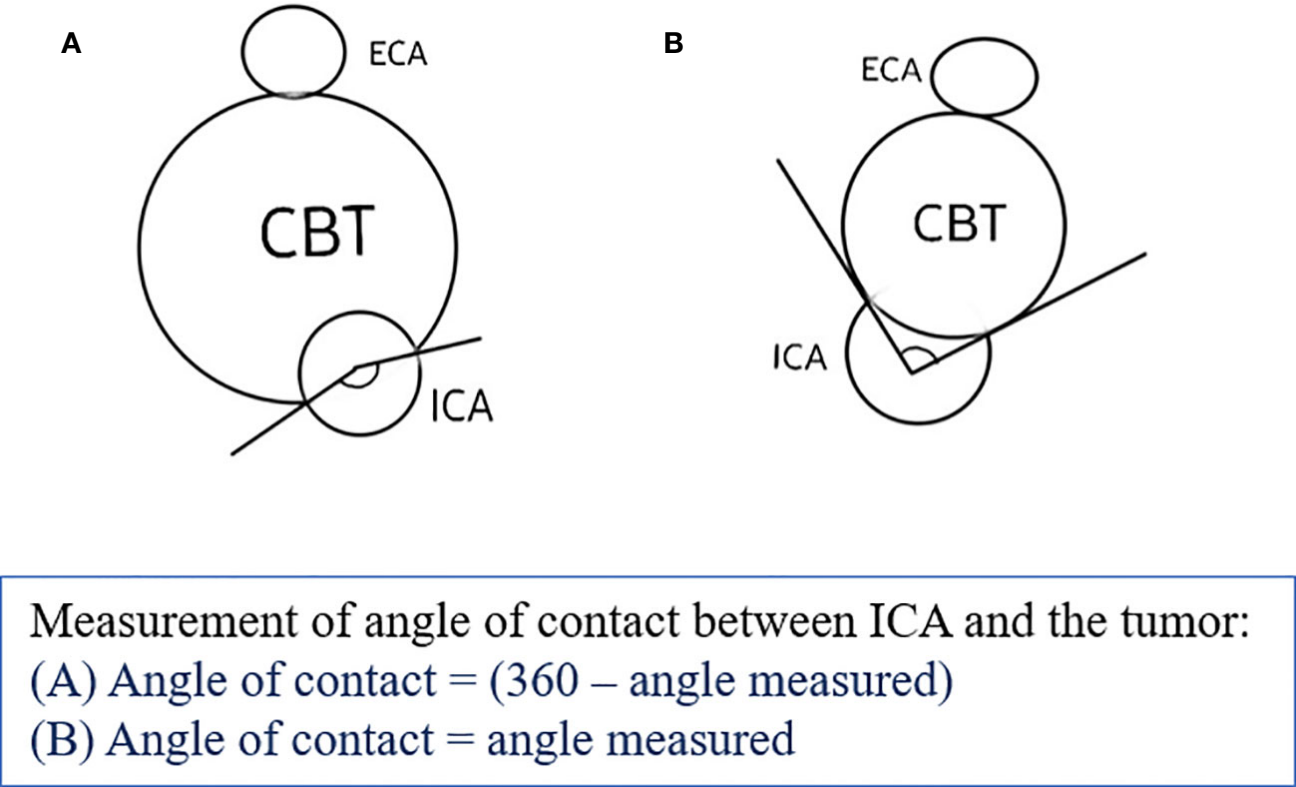
Schematic diagram showing measurement of the angle of contact of the tumor with the ICA.

The study was approved by the institutional review board. We retrospectively reviewed the electronic medical records (EMR) of patients registered in tertiary cancer care hospitals from 1 January 2004 to 30 November 2020. After institutional ethics committee approval, eligible patients were included in the study after applying the inclusion and exclusion criteria. The inclusion criteria in our study were patients who had a primary diagnosis of carotid body tumor, either histopathologically proven or consensually diagnosed by a multidisciplinary tumor board, and had MR imaging available for review (minimum T1, T2, STIR, and postcontrast T1 sequences). Patients with no follow-up data or required MR sequences were excluded. The baseline demographic details were recorded using the patient’s EMR. MRI features of each patient from the hospital Picture Archiving and Communication System (PACS) database were assessed by two blinded radiologists. The three MRI scanners used were Philips Ingenia 1.5 T, GE Signa 1.5 T, and GE Signa 3 T. A dedicated neck surface coil was used to obtain sequences, acquired at 4-mm thickness with no intersection gap. Postcontrast studies were performed after injection of 0.1 mmol/kg of gadolinium-diethylenetriaminepentaacetic acid. In cases of MR angiography, 2D time-of-flight (TOF) or 3D TOF sequences, with a section thickness of 1 mm were taken. Pretreatment MRI of patients with the following sequences was assessed: T1, T2, short tau inversion recovery (STIR), T1 postcontrast, and diffusion-weighted imaging (DWI) sequences in multiple planes. The patient’s EMR data were used to obtain data regarding clinical symptoms, surgical, histopathological, and radiation therapy details, as well as relevant follow-up data. Entries were made appropriately as per discussion with the radiologists. MR images were reviewed for tumor characteristics on T1, T2, DWI, and postcontrast T1 sequences along with the site, size, laterality of tumor, presence of peritumoral tufts of veins, loss of fat planes with adventitia of carotid vessels, distance from skull base, and involvement of carotid vessel including angle of contact. Shamblin, Modified Shamblin, and PUMCH grades were designated for the tumors. We reviewed these findings in our study to propose a new grading system, namely the Mahajan et al. grading system based on the distance from the skull base and angle of contact with the internal carotid artery (ICA), as described in **Table 1**. The distance from the skull base was calculated as the maximum distance from the superior margin of the tumor to the jugular foramen on the sagittal or coronal MR sequence, and the angle of contact with ICA was measured on the axial MR sequence (**Figure 1**). These features were decided after a review of the literature by Shamblin et al. (14), Luna-Ortiz et al. (15, 16), Gu et al. (18), Jasper et al. (19), and Arya et al. (20).

**Table 1:** Mahajan et al grading of carotid body tumor.

Grade	Distance from skull base (cm)	Angle of contact with ICA (degrees)
I	> 6	< = 180
II	> 6	181 – 270
	3.1 – 6	<= 180
IIIa	3.1 – 6	181 – 270
IIIb	< 3	> 270

A score was also given on the basis of MR characteristics of tumors that influence the management decision. It was correlated with intra- and postoperative findings from the available EMR data. A change in tumor size was analyzed in patients undergoing radiation therapy or conservative management using follow-up MRI and EMR details. Data were organized in proper format, and statistical analysis was done.

### Risk score

On the basis of the MRI characteristics of the tumor, scores were designated as mentioned in **Table 2**, and the total score was calculated for every patient. The cut-offs were designated after reviewing the literature and imaging of patients at our institute. It was then tested statistically using ROC to verify our hypothesis, as represented in **Table 3**. The variables considered were the angle of contact, tumor size, tumor volume, tumor characteristics on T2, STIR, and postcontrast MRI sequences, the presence of peritumoral veins, and loss of fat planes with adventitia of carotid vessel wall. These variables were compared for distance from the skull base and angle of contact separately. The cut-off for distance from the skull base below which the tumor showed high surgical risk was then taken as 3 cm using ROC. The angle of contact showed comparatively greater dispersion of values; however, to keep it universal and simple, values of 180 and 270 were taken for cut-off.

**Table 2:** Risk score for surgery in carotid body tumors.

Score	0	1	2
Tumor Volume (cc)	<= 15	16 – 30	> 30
Tumor size (cm)	<= 3	3.1 – 6	> 6
Laterality	Unilateral	Bilateral	
Distance from skull base	> 3	< = 3	
Location in neck	Below Angle of Mandible	Above Angle of Mandible	Above Mastoid tip
Angle of contact with ICA	<= 180	181 – 270	>= 270
T2W	Hyperintense	Heterogenous	
DWI	Non restricted	Restricted	
Postcontrast enhancement	Homogeneous	Heterogeneous	
Peritumoral veins	Absent	Present	
Loss of fat planes with vessel wall	Absent	Present	
Total Score			

**Table 3:** Table showing a comparison of angle of contact and tumor size against tumor characteristics.

	AUC	Cut-off	Specificity	Sensitivity
Tumor size				
Loss fat planes adventitia	0.9773	2.7750	1.0000	0.8500
Peritumoral veins	0.9898	2.6250	1.0000	0.9000
T2/STIR	0.8032	3.8000	0.8065	0.6500
Postcontrast	0.8339	3.1250	0.6563	0.8421
DFSB	0.7135	3.1250	0.6429	0.7391
Angle of contact (≥ 270)	0.7940	3.0250	0.6129	0.9000
Tumor volume (> 30)	0.9910	3.5250	1.0000	0.9565
Angle of contact				
Loss fat planes adventitia	0.8761	156.50	0.9091	0.8250
Peritumoral veins	0.8102	156.50	0.8182	0.8000
T2/STIR	0.7387	211.00	0.6774	0.8000
Postcontrast	0.7722	211.00	0.6875	0.8421
Tumor size (> 3)	0.8100	144.00	0.5652	0.9643
Tumor volume (> 30)	0.7880	211.00	0.7143	0.7826

### Statistical analysis

The patient’s demographics, treatment, and outcome data were entered, and all statistical analyses were performed using IBM SPSS software v25. Data were summarized and descriptively analyzed using frequency and percentage for categorical data. Chi-square, Fischer’s exact test, and Pearson correlation coefficient were used to observe the association and correlation between the two variables. The area under the curve (AUC) and ROC curves were used to present the accuracy of different predictive models. Cut-offs for ROC were calculated using the Youden index method. All statistics were two-sided, and a *p*-value of <0.05 was considered statistically significant. The sensitivity and specificity were obtained by calculating the area under the curve using ROC. The ROC curve was constructed for the total score to select cut-off values for different classifications.

## Results

A total of 44 patients with 51 lesions were included in the study, and analysis of individual lesions was done. The demographic distribution showed no gender predilection in patients studied at our institute, and the age of the patients ranged from 11 to 73 years at the time of presentation. Out of 44 patients, 37 patients had unilateral lesions, and the rest, seven patients, had bilateral lesions. Clinically, most patients presented with painless swelling, accounting for almost 61%, while the remaining patients presented with symptoms of pain, dysphagia, or hoarseness.

MRI review of the CBTs included in our study showed characteristics described in **Table 4**. The tumor’s mean size was 3.4 cm in maximum axial dimension, with the mean volume of tumor being 40 cm^3^. The mean distance from the skull base was 3.6 cm, measured craniocaudally. In total, 76% were located above the angle of mandible and below the mastoid tip. While 22% of tumors had maintained fat planes, 78% showed loss of fat planes with adjacent carotid vessel wall adventitia. Peritumoral veins were also present in about 78% of tumors and showed a similar distribution as loss of fat planes with carotid vessels, likely correlating with tumor extension. The majority (82%) of tumors showed isointensity on T1W as compared to muscle. Two (4%) lesions showed hyperintensity on T1, likely due to intratumoral bleed, and seven (14%) lesions were hypointense, suggesting fibrotic changes in the tumor. T2W images showed homogenous hyperintensity in 61% of tumors, and 39% of tumors being heterogeneous on T2, suggesting fibrosis in the lesions. STIR sequences showed hyperintensity in 61% of lesions, while the remaining appeared heterogeneous and showed a correlation with T2 characteristics. Out of 51 lesions, DWI was available for only 33 lesions for review. Of the 33 lesions, 29 (88%) showed diffusion restriction, indicating high cellularity, while the rest showed no restriction. On administration of gadolinium intravenously, acquisition of T1 sequences showed homogenous enhancement in 63% of tumors, with the rest showing heterogeneous postcontrast enhancement, correlating with STIR and T2 characteristics and indicating fibrosis in the tumor. The angle of contact of CBT with ICA was measured on axial sequences (20). All 51 lesions were classified into three available grading systems based on MRI characteristics, including the new proposed grading system by Mahajan et al., as presented in **Table 5**.

**Table 4:** MRI characteristics of tumors included in the study.

Variable	Frequency	Percentage
Size (cm)		
≤ 3	21	41.2
3–6	28	54.9
> 6	2	3.9
Volume (cm^3^)		
≤ 15	17	33.3
16–30	11	21.6
> 30	23	45.1
Distance from skull base (cm)		
> 6	5	9.8
3.1–6	23	45.1
≤ 3	23	45.1
Location		
Below the angle of mandible	9	17.6
Above the angle of mandible	39	76.5
Above the mastoid tip	3	5.9
Planes with vessel adventitia		
Absent	11	21.6
Present	40	78.4
Peritumoral veins		
Absent	11	21.6
Present	40	78.4
T1		
Hyperintense	2	3.9
Isointense	42	82.4
Hypointense	7	13.7
T2		
Hyperintense	31	60.8
Heterogenous	20	39.2
STIR		
Hyperintense	31	60.8
Heterogenous	20	39.2
Postcontrast enhancement		
Homogeneous	32	62.7
Heterogeneous	19	37.3
DWI		
Restriction	29	56.9
No restriction	4	7.8
Not available	18	35.3
Angle of contact		
≤ 180	22	43.1
180–270	9	17.6
≥ 270	20	39.2

**Table 5:** Classifications of carotid body tumor.

Variable	Frequency	Percentage
Shamblin et al. (14)		
I	22	43.1
II	9	17.6
III	20	39.2
Luna-Ortiz et al. (15)		
I	10	19.6
II	0	0
IIIa	1	2.0
IIIb	40	78.4
Peking Union Medical College Hospital (18)		
I	9	17.6
II	15	29.4
III	9	17.6
IV	14	27.5
V	4	7.8
Mahajan et al.		
I	5	9.8
II	10	19.6
IIIa	4	7.8
IIIb	32	62.7

### Risk score calculation

The risk score was calculated using the variables and scoring described in **Table 2**. The total score was 15, with a maximum calculated score of 13 and a minimum score of 0 for patients in the study. The cut-off score was calculated for each classification using ROC. Tumors with a score of 0–6 were regarded as low risk, and tumors with a score of 7–15 were regarded as high risk for surgery. The frequency of vascular injury and cranial nerve injury in each category of various classifications is enumerated in **Table 6**. However, numbers are small to statistically prove the superiority of one classification over the other. Numerous tumor features impact the outcomes of these tumors, and Mahajan et al. comprehensively incorporate these factors in their classification. However, the classification needs to be validated with a larger number of patients.

**Table 6:** Table showing a comparison of high- and low-risk scores with various classifications.

Classification	0 to 6 (Low risk)	7 to 15 (High risk)	Operated	Vascular Injury	Cranial Nerve Injury
Shamblin					
I	18 (78%)	4 (14%)	5/22 (23%)	0	3/5 (60%)
II	4 (17%)	5 (18%)	5/9 (55%)	4/5 (80%)	1/5 (20%)
III (High risk)	1 (5%)	19 (68%)	6/20 (30%)	4/6 (66%)	2/6 (33%)
Luna Ortiz					
I	10 (43%)	0	3/10 (30%)	0	1/3 (33%)
II	0	0	0	0	0
IIIa	1 (4%)	0	0	0	0
IIIb	12 (52%)	28 (100%)	13/40 (33%)	8/13 (61%)	5/13 (38%)
PUMCH					
I	7 (21%)	2 (7%)	2/9 (22%)	1/2 (50%)	1/2 (50%)
II	12 (36%)	3 (11%)	4/15 (27%)	0	2/4 (50%)
III	4 (12%)	5 (18%)	5/9 (55%)	4/5 (80%)	1/5 (20%)
IV	0	14 (50%)	5/14 (36%)	3/5 (60%)	2/5 (40%)
V	0	4 (14%)	0	0	0
Mahajan					
I	4 (17%)	1 (3%)	2/5 (12%)	1/2 (50%)	1/2 (50%)
II	10 (44%)	0 (0%)	3/10 (30%)	0	2/3 (66%)
IIIa (High risk)	4 (17%)	0 (0%)	0	0	0
IIIb (High risk)	5 (22%)	27 (97%)	11/16 (69%)	7/11 (64%)	3/11 (27%)

### Management characteristics

Out of 44 patients with CBTs presented at our institute, seven had bilateral and 37 had unilateral tumors. For bilateral tumors, three (43%) were operated on for one side, and the second lesion was either kept under observation or planned for radiotherapy (RT); two patients were kept on observation for both lesions, and the remaining two were lost to follow-up. For unilateral tumors, 13 (35%) patients underwent surgery, and four underwent RT. RT was given to patients with large, unresectable tumors encasing the ICA. Of the 16 patients who underwent surgery, 12 (75%) had a complete resection of the tumor, while four underwent debulking. Among patients who underwent surgery, five patients were Shamblin I and II each, and six patients were Shamblin III. Among patients who underwent surgery, two belonged to grade I, three belonged to grade II, and the remaining 11 belonged to grade IIIb of the Mahajan classification. Major complications associated with surgery in CBTs include hemorrhage, stroke, cranial nerve injury, and the need for vascular repair and/or reconstruction. Of the patients who underwent surgery at our institute, eight had to undergo vascular repair. Among patients who underwent vascular repair, tumors belong to Shamblin II (50%) and III (50%); Luna-Ortiz class IIIb (100%); PUMCH classes I (12.5%), III (50%), and IV (37.5%); and Mahajan classes I (12.5%) and IIIb (87.5%). Two patients had documented hemorrhage during surgery amounting to 800–1,000 mL of blood loss, and three patients had a history of stroke postsurgery. Six patients had cranial nerve (CN) injury, most commonly CN X, the others being VII, IX, and XII, belonging to Mahajan classes II and IIIb.

### Follow-up characteristics

Follow-up MRIs were available for 10 patients in our study and showed around a 1% average increase in the size of tumors in a median follow-up period of 25 months. Some patients could not be followed up beyond 1 month, while others were followed up to 32 months.

## Discussion

CBTs are rare but the most common paragangliomas of the head and neck (3). Surgery is the curative treatment, requiring complete excision of the tumor with preservation of surrounding vital neurovascular structures (21). Radiation therapy is another treatment option, but it is reserved for tumors that are unresectable or multiple in number (12). The principal factors affecting the surgical treatment of CBTs apart from age and operative risk are multifocality and the possibility of impairment of cranial nerves and injury to adjacent carotid vessels (11). In our study at a tertiary cancer care hospital, out of 44 patients with 51 lesions, 16 underwent surgical excision, five underwent radiotherapy, and the rest were observed. A follow-up MRI was available for 10 patients. Being a rare tumor, the availability of literature, large cohort studies, and case series was limited. We have compared it with some popular and recent available literature, as represented in **Table 7**.

**Table 7:** Comparison of studies on carotid body tumors.

	Present study	Shamblin et al. (14)	Luna-Ortiz et al. (15)	Gu et al. (18)	Jasper A et al. (19)	Amr Gad et al. (22)	Prasad et al. (23)
**Duration of study (years)**	16	36	22	7	13	25	26
**Sample size**	44	90	49	105	40	56	20
**Mean age (years)**	41	40	47	43	39	42	43.8
**Minimum age (years)**	11	12	18	20	20	32	17
**Maximum age (years)**	73	63	73	71	67	47	60
**Male**	22 (50%)	62 (69%)	48 (98%)	35 (33%)	22 (55%)	39 (70%)	8 (42%)
**Female**	22 (50%)	28 (31%)	1 (2%)	70 (67%)	18 (45%)	17 (30%)	11 (58%)
**Unilateral**	37 (84%)	89 (99%)	48 (98%)	84 (80%)	38 (95%)	54 (96%)	14 (70%)
**Bilateral**	7 (16%)	1 (1%)	1 (2%)	21 (20%)	2 (5%)	2 (4%)	6 (30%)
**Shamblin I**	22 (43%)	23 (26%)	8 (16%)	27 (23%)	6 (14%)	22 (40%)	5 (18%)
**Shamblin II**	9 (18%)	42 (46%)	17 (34%)	23 (20%)	15 (36%)	26 (46%)	15 (53%)
**Shamblin III**	20 (39%)	25 (27%)	24 (49%)	66 (57%)	21 (50%)	8 (14%)	8 (28%)
**Surgery**	16 (36%)	57 (63%)	49 (100%)	105 (100%)	40 (100%)	56 (100%)	24 (86%)
**Radiation**	5 (11%)	11 (12%)	0	0	0	0	NA

NA, data not available.

The clinical presentation showed a similar distribution among various studies. Using cross-tables, the other three classifications were correlated with the Shamblin classification. Modified Shamblin by Luna-Ortiz et al. showed an upgradation of 12 class I tumors to IIIb due to tumors showing loss of fat planes with carotid vessel adventitia. The remaining 29 tumors showed the same grade as the original Shamblin class. PUMCH by Gu et al. showed 22 Shamblin I tumors distributed in types I, II, and V; Shamblin II tumors in type III; and Shamblin III tumors in types I, IV, and V. The irregular distribution can be attributed to the location of the tumor evaluated in the PUMCH classification, which was not included by Shamblin. Similar upgradation is also seen in the Mahajan classification, with four grade I, 10 grade II, and eight grade IIIb tumors belonging to Shamblin I. Out of nine Shamblin II tumors, one belonged to grade I, four to grade IIIa, and four to grade IIIb, whereas all 20 Shamblin III tumors belonged to Mahajan IIIb. The location of the tumor considered in the PUMCH classification indirectly correlates with the distance of the tumor from the skull base, while vessel wall infiltration considered by Luna-Ortiz indirectly correlates with the angle of contact. These features estimate the surgical risk and perioperative morbidity associated with the management of CBTs. Various tumor factors impact the surgical morbidity of these tumors, and the Mahajan classification comprehensively includes these factors.

MRI evaluation of CBT has proved to be immensely helpful in diagnosis, management, and prognostication. The typical MRI findings include a hyperintense mass on T2 with flow voids splaying the carotid bifurcation (24, 25). Postcontrast sequences show early-phase arterial enhancement. Apart from these typical MRI findings, additional sequences like DWI and dynamic sequences are also useful for the differentiation of CBTs from other lesions in the head and neck (24). MRI also provides the angle of contact of the tumor with ICA, which is imperative for the decision of surgery and forms the basis of the surgical classification of CBTs as described by Arya et al. in their study (20). Measurement of distance from the skull base also helps to evaluate the risk of injury to vital neurovascular structures, as demonstrated by Luna-Ortiz et al. (16) and Gu et al. (18) in their studies. The presence of peritumoral veins and the loss of fat planes with adventitia of vessel wall have been put forth by Jasper et al. for the prediction of the risk of hemorrhage (19). The recent study by Prasad et al. also proposes the importance of the angle of contact of the tumor with carotid vessels and its location in parapharyngeal space. Their classification takes into account the involvement of the carotid arteries and the compartmentalization of the tumor into upper, middle, and lower parapharyngeal spaces. The choice of intraarterial stenting and surgical approach is determined according to the infiltration of the artery and the extent of the tumor according to these compartments (23).

The new scoring system for predicting risk in the surgical management of CBTs allots a score of 0 to 15 on the basis of MRI, as discussed earlier. A score of 0–6 showed low risk, while 7–15 showed high risk for surgery. A scoring system based on CT features of tumor volume, angle of contact, presence of peritumoral veins, and loss of tumor adventitial surface was proposed by Jasper et al. (19).

In total, 16 (36%) lesions among 44 patients underwent surgical excision of CBT at our institute; 12 of them underwent complete excision, and three patients underwent incomplete excision with only debulking of the tumor. Surgery for one patient was abandoned due to extensive involvement of carotid vessels and was later planned for radiotherapy. Eight (50%) patients had vascular ligation and reconstruction. Cranial nerve injury and subsequent palsy were seen in six (37%) patients. These patients belonged to Shamblin II (50%) and III (50%); Luna-Ortiz IIIb (100%); PUMCH I (12.5%), III (50%), and IV (37.5%); and Mahajan I (12.5%) and IIIb (87.5%). Cranial nerve injury was seen to occur in all classes of tumors without any predilection in our sample of patients. Follow-up MRIs were available for 10 patients, with a median follow-up period of 25 months. The tumor size and volume of patients on observation remained largely unchanged. Similar findings were also noted in the study by Shamblin et al.

The strengths of the study include a single, large cohort study of a rare tumor using essential imaging features to predict the surgical outcome. We have also proposed a scoring system for the assessment of perioperative risks using a noninvasive imaging technique and a modified classification based on various tumor factors for better stratification of carotid body tumors for surgical risk assessment. Limitations include the retrospective nature of the study and the limited number of patients who underwent surgery. A long-term follow-up with a 5-year survival rate for this indolent tumor was not available. Given the rarity of the disease, a multicenter, large cohort study needs to be carried out in a prospective manner to validate the clinical applicability of the proposed classification and scoring system.

## Conclusion

Though the Shamblin classification of CBT is the most widely accepted classification, our proposed Mahajan classification system provides an imaging-based alternative to prognosticate surgical candidates preoperatively. The Mahajan classification of CBTs evaluates high-risk factors like the distance of the tumor from the skull base and the angle of contact with ICA, which form the major predictors of neurovascular damage and morbidity associated with its surgery. Our proposed MRI-based Mahajan classification and risk scoring system can be used as an effective prognostication tool to preoperatively stratify high- and low-risk surgical candidates.

## Data availability statement

The raw data supporting the conclusions of this article will be made available by the authors, without undue reservation.

## Author contributions

AM and AS conceived and designed the structure of this manuscript. AM, AS and SSh wrote the paper. AM, AS, SSh and UA revised the paper. All authors contributed to the article and approved the submitted version.
